# Gene–physical activity interactions in lower extremity performance: inflammatory genes CRP, TNF-α, and LTA in community-dwelling elders

**DOI:** 10.1038/s41598-017-03077-1

**Published:** 2017-06-15

**Authors:** Chiu-Shong Liu, Tsai-Chung Li, Chia-Ing Li, Li-Na Liao, Chuan-Wei Yang, Chih-Hsueh Lin, Nai-Hsin Meng, Wen-Yuan Lin, Sung-Lin Hu, Jen-Hao Hsiao, Fang-Yang Wu, Cheng-Chieh Lin

**Affiliations:** 1 School of Medicine, College of Medicine, China Medical University, Taichung, Taiwan; 2 Department of Family Medicine, China Medical University Hospital, Taichung, Taiwan; 3 Department of Public Health, College of Public Health, China Medical University, Taichung, Taiwan; 4 Department of Healthcare Administration, College of Medical and Health Sciences, Asia University, Taichung, Taiwan; 5 Department of Medical Research, China Medical University Hospital, Taichung, Taiwan; 6 Ph.D. Program for Aging, College of Medicine, China Medical University, Taichung, Taiwan; 7 Department of Physical Medicine and Rehabilitation, China Medical University Hospital, Taichung, Taiwan; 8 Bioinformatics and Biostatistics Core, Center of Genomic Medicine, National Taiwan University, Taipei, Taiwan

## Abstract

We assessed gene–gene and gene-physical activity interactions of polymorphisms in C-reactive protein (CRP), tumor necrosis factor-α (TNF-α), and lymphotoxin α (LTA) genes on lower extremity performance in community-dwelling elders in Taiwan. Five SNPs (rs1205, rs1130864, rs1800947, rs2794520, and rs3093059) of CRP gene, three SNPs (rs909253, rs1041981, and rs2239704) of LTA gene, and three SNPs (rs3093662, rs1800629, and rs1799964) of TNF-α gene of 472 unrelated elders were genotyped. Lower extremity performance included timed up-and-go test (TUG), walking speed, weight-adjusted leg press (waLP), and timed chair stand (TCS). We detected significant interactions between physical activity with CRP rs2794520, rs1205, and rs3093059; LTA rs909253 and rs1041981; and TNF-α rs1799964 for TCS in women after covariate adjustment (all P < 0.05). In men, significant interactions between physical activity with CRP rs2794520, rs1205, and rs3093059; and LTA rs909253 and rs1041981 for TUG; with CRP rs2794520, rs1205, rs1130864, and rs3093059; and LTA rs909253 and rs1041981 for walking speed; and with TNF-α rs3093662 for waLP after covariate adjustment (all P < 0.05). These variants also significantly interacted with physical activity on TCS in women and on walking speed in men. These results show inflammatory genes are involved in lower extremity performance, likely via gene–physical activity interactions.

## Introduction

Aging is associated with reduced physical function, which may lead to an inability to perform normal daily activities^1^. Age-related decline in the physical function of the elderly can lead to frailty and loss of independence. Physical dysfunction is associated with adverse health outcomes, such as nursing home admissions, mortality, falls, and hospitalization^2–4^.

Inflammation and cytokine activation play a critical role in the aging process. Systemic chronic inflammatory state is associated with all-cause mortality in the elderly^5, 6^ and many aged-related diseases, such as coronary heart disease^7^, Alzheimer’s disease^8^, and osteoporosis^9^, which may result from tissue damage caused by chronic, low-grade inflammatory state. Lines of evidence support inflammation links with bone and muscle loss, anemia, and insulin resistance with a network of interacting cytokines, including tumor necrosis factor (TNF)-α^10^ and C-reactive protein (CRP)^11^. Elevation in the inflammatory cytokines level is considered a predictor of sarcopenia^12^ and is associated with reduced muscle strength^13^ and physical performance^14^. Several studies have supported an important role of inflammation in frailty observed in cross-sectional studies, and significant positive relationships were identified between frailty and inflammatory cytokines, such as CRP^11^.

Genetic variations play a critical role in the inflammation process. Chronic, low-grade inflammatory state has been found to interact with genetic constitution to act on death^5, 15^ and some aged-related diseases, such as Alzheimer’s disease^16^, cardiovascular disease^17^, atherosclerosis^18^, and type-2 diabetes^19^. Genetic constitution involved in systemic inflammation may alter the intensity of immune responses and influence the susceptibility to these age-related diseases by increasing gene transcription and cytokine production or decreasing proinflammatory cytokine production^20, 21^. Genetic factors may affect the physical function of the elderly^22^, and the heritability of muscle strength varies from 36% to 65%^23–25^. These studies have thus suggested that inflammatory response is involved in the association between physical function or performance and gene polymorphisms^26^.

TNF-α and lymphotoxin α (LTA; also known as TNF-β) genes, a cluster of cytokines being located next to each other in chromosome 6p21.3 that harbors the class III region of the major histocompatibility complex locus^27^, belong to the TNF superfamily. Both TNF-α and LTA have similar effects and common receptors^28^. Polymorphisms in the LTA gene had been associated with the production of serum TNF-α^29^. Previous studies on the link between expression and disease mainly on TNF-α. TNF-α is one of the proinflammatory cytokines and plays a critical role in initiating and regulating the cytokine cascade during an inflammation response^30^. Prior animal studies showed that TNF-α also plays a central role on restricting the degree and duration of an inflammation by promoting repair and recovery from infectious and toxic agents^30^. Little or no initial response to infectious challenge was found under TNF-α-deficient state, resulting in a thriving and disorganized inflammatory response. Initiation of the inflammatory cascade would induce liver-produced acute-phase proteins, such as CRP^20^.

CRP, an acute-phase protein synthesized primarily by the liver, is a phylogenetically ancient regulator mapped in 1q23.2^31^. CRP is also a heritable marker of chronic inflammation strongly associated with cardiovascular risk factors, such as age, gender, ethnicity, body mass index (BMI), adiposity, hyperinsulinemia, and insulin resistance^32^, and increased risks of cardiovascular morbidity and mortality^33^. Several polymorphisms of the CRP gene were associated with plasma CRP concentration^34^, but these polymorphisms account for a portion of the reported overall 35% to 50% heritability^35, 36^. A genome-wide association study found that the most influential of the polymorphisms in CRP gene was rs3091244 (T and A alleles). The other polymorphisms identified by the other studies were rs1205 (G allele), rs1130864 (T allele), and rs3093075 (C allele)^37–39^. SNP rs2794521 (T allele) has been reported to upregulate the transcription of the CRP allele^40^. Lower CRP levels have been linked with the C allele of SNP rs1800947^37, 40^, and common haplotypes of the gene are also correlated with 2–3-fold increases in serum CRP concentration^38, 40^. Eleven common SNPs with minor allele frequency >5% within 6 kb of the CRP gene (HGNC: 2637; 1q21-q23) were found by a study with participants of European descent, but extensive linkage disequilibrium (LD) indicates that four major haplotypes account for 94% of chromosomes^41, 42^.

Several epidemiologic studies have explored the relationship between inflammation markers and a decline in muscular mass and strength^14, 43–45^. However, genetic studies on the association between inflammatory genetic markers and muscular mass and strength are limited. We previously detected that SNP rs2794520, rs1205, and rs3093059 of the CRP gene were significant biomarkers for susceptibility to low handgrip strength and high serum CRP level in Chinese elders residing in a community^42^. Significant interactions of TNF-α rs1799964 and LTA rs909253 on handgrip strength were also observed^46^. In addition, remarkable joint effects of the CRP, TNF-α, and LTA risk alleles with physical inactivity on handgrip strength were noted. Associations between polymorphisms in the promoter region of the TNF-α coding gene and muscle phenotypes had been reported^47^. However, few studies tested the effect of inflammation genes on the lower extremity performance, such as walking speed and standing up from a 16-in chair. In addition, the effects of single-gene polymorphism on physical performance may be masked by gene–gene and gene–environment interactions. Physical exercise may attenuate age-related chronic inflammation and improve physical performance^26^. Thus, the interaction of the genes on the same pathway and of gene–physical activity should be explored to elucidate actual influences on physical performance. The present study investigated five tag-SNPs (rs1205, rs1130864, rs1800947, rs2794520, and rs3093059) in the CRP locus and six common genetic variants (rs3093662, rs1800629, and rs1799964; rs909253, rs1041981, and rs2239704) in the TNF-LTA locus to test whether these variants interacted with each and with physical activity for lower extremity performance. We hypothesized that the genetic variants of CRP, TNF-α, and LTA are associated with lower extremity performance in the elderly by haplotype effects and gene–gene and gene–physical activity interactions.

## Results

The characteristics of elders in this study are shown in Table 1. The mean age of the community-dwelling male and female elders was 72.8 ± 5.5 and 74.7 ± 6.4 years, respectively. Among these elders, 53.2% were men. The self-reported diagnosed diseases included hypertension (47.4%), hyperlipidemia (25.7%), diabetes (13.9%), cancer (5.5%), and stroke (4.6%). Men displayed higher prevalence of physical activity, smoking habits, and alcohol drinking habits than women (P = 0.038, <0.001, and <0.001, respectively). The mean values of weight-adjusted leg press (waLP) and TNF-α level were higher in men than in women (P < 0.001 and 0.008, respectively). There are no difference in distributions of BMI, walking speed, timed up-and-go (TUG), timed chair stands (TCS), status for currently being employed, protein intake, fruit or vegetable intake, hs-CRP, stroke, cancer, and number of disease history.

**Table 1:** Characteristics of study subjects.

Characteristic	Total (n = 472)	Women (n = 221, 46.8%)	Men (n = 251, 53.2%)	P-value^d^
Age (year)	73.8 ± 6.1	72.8 ± 5.5	74.7 ± 6.4	0.001
BMI (kg/m^2^)	23.5 ± 3.2	23.5 ± 3.3	23.5 ± 3.2	0.829
**Physical performance**				
Walking speed (m/sec)	0.9 ± 0.2	0.8 ± 0.2	0.9 ± 0.2	0.082
Timed up and go (sec)	7.9 ± 4.8	8.0 ± 4.8	7.9 ± 4.8	0.840
WaLP (%)	93.9 ± 38.1	82.6 ± 36.0	103.7 ± 37.2	<0.001
Timed chair stands (sec)	5.4 ± 3.0	5.5 ± 2.8	5.2 ± 3.1	0.391
**Behavior**				
Physical activity	376 (79.7)	167 (75.6)	209 (83.3)	0.038
Smoking	100 (21.2)	7 (3.2)	93 (37.1)	<0.001
Alcohol drinking	94 (19.9)	14 (6.3)	80 (31.9)	<0.001
Currently employed	51 (11.33)	18 (8.61)	33 (13.69)	0.122
Diet				
Protein intake^a^				0.258
0	82 (17.41)	43 (19.55)	39 (15.54)	
0.5	241 (51.17)	104 (47.27)	137 (54.58)	
1	148 (31.42)	73 (33.18)	75 (29.88)	
Fruit or vegetable intake^b^	421 (90.34)	196 (89.91)	225 (90.73)	0.888
**Biochemical marker**				
hs-CRP (mg/L)^c^	0.1 ± 3.2	0.1 ± 3.2	0.1 ± 3.2	0.884
TNF-α (pg/mL)^c^	1.6 ± 4.0	1.3 ± 4.0	1.8 ± 3.9	0.008
**Disease history**				
Hypertension	221 (47.4)	98 (45.2)	123 (49.4)	0.003
Diabetes	65 (13.9)	21 (9.6)	44 (17.8)	0.010
Hyperlipidemia	118 (25.7)	70 (32.1)	48 (19.8)	0.003
CVA	21 (4.6)	10 (4.6)	11 (4.5)	0.975
Cancer	25 (5.5)	13 (6.1)	12 (5.0)	0.615
Number of disease history				0.834
0	166 (35.32)	77 (34.84)	89 (35.74)	
1	191 (40.64)	89 (40.27)	102 (40.96)	
2	83 (17.66)	43 (19.46)	40 (16.06)	
3	27 (5.74)	11 (4.98)	16 (6.43)	
4	3 (0.64)	1 (0.45)	2 (0.80)	

Data are presented as mean ± SD for continuous variables or n (%) for categorical variables.

BMI: body mass index; hs-CRP: high-sensitivity C-reactive protein; CVA: cerebral vascular accident.

a: Mini nutritional assessment (MNA) questionnaire: selected consumption markers for protein intake, including (1) at least 1 serving of dairy products per day, (2) two or more servings of legumes or eggs per week, and (3) meat, fish or poultry every day. 0 = if 0 or 1 yes, 0.5 = if 2 yes, and 1 = if 3 yes.

b: MNA questionnaire: consumes two or more servings of fruit or vegetables per day.

c: Geometric mean was presented.

d: Two-sample t-test for continuous variables or χ^2^-test for categorical variables.

All 11 SNPs studied in the three genes were within the Hardy–Weinberg equilibrium (HWE) (P > 0.05), and the allele and genotype distributions for all polymorphisms were similar across sex (P > 0.05) (Table 2). The mean values of walking speed, TUG test, waLP, and TCS test for the genotypes of CRP and TNF/LTA polymorphisms were described separately according to gender. We did not observe significant main effects of these polymorphisms on these four outcomes. We detected significant relationship between serum CRP levels and these four outcomes both in men and women. Spearman correlation coefficients for log-transformed CRP and TUG, walking speed, waLP, and TCS in women were 0.24 (P < 0.01), −0.23 (P < 0.001), −0.13 (P < 0.06), and 0.16 (P < 0.05), respectively. The corresponding values for men were 0.18 (P < 0.01), −0.19 (P < 0.01), −0.19 (P < 0.01), and 0.16 (P < 0.05).

**Table 2:** Genotype and allele distributions of study subjects stratified by sex^a^.

SNP (Gene)	Genotype or allele	Frequency n (%)	Walking speed (m/sec)	TUG (sec)	WaLP (%)	Chair rise 3 (sec)
**Women**						
rs2794520	AA	71 (32.1)	0.86 ± 0.23	8.31 ± 7.28	86.52 ± 39.18	5.64 ± 3.92
(CRP)	GA	109 (49.3)	0.83 ± 0.18	7.70 ± 2.98	79.80 ± 33.67	5.35 ± 1.93
	GG	41 (18.6)	0.80 ± 0.24	8.21 ± 3.11	83.57 ± 36.74	5.61 ± 2.41
	G*	191 (43.2)				
rs1205	AA	71 (32.3)	0.86 ± 0.23	8.31 ± 7.28	86.52 ± 39.18	5.64 ± 3.92
(CRP)	GA	110 (50.0)	0.83 ± 0.18	7.69 ± 2.97	79.52 ± 33.63	5.34 ± 1.92
	GG	39 (17.7)	0.79 ± 0.24	8.32 ± 3.16	85.54 ± 37.06	5.64 ± 2.48
	G*	188 (42.7)				
rs1130864	GG	194 (88.2)	0.85 ± 0.21	7.85 ± 4.79	84.65 ± 36.72	5.33 ± 2.63
(CRP)	AG	26 (11.8)	0.76 ± 0.21	9.06 ± 4.82	68.79 ± 27.12	6.66 ± 3.62
	AA	0 (0.0)	—	—	—	—
	A*	26 (5.9)				
rs1800947	CC	181 (81.9)	0.83 ± 0.21	8.08 ± 5.01	81.11 ± 36.04	5.56 ± 2.89
(CRP)	GC	37 (16.7)	0.89 ± 0.20	7.50 ± 3.79	93.37 ± 32.82	4.95 ± 2.10
	GG	3 (1.4)	0.72 ± 0.15	8.52 ± 1.62	43.16 ± 40.05	7.56 ± 3.25
	G*	43 (9.7)				
rs3093059	AA	142 (64.3)	0.84 ± 0.21	8.13 ± 5.68	80.93 ± 35.43	5.61 ± 3.25
(CRP)	GA	68 (30.8)	0.82 ± 0.19	7.76 ± 2.30	85.14 ± 38.02	5.29 ± 1.63
	GG	11 (5.0)	0.84 ± 0.29	7.43 ± 2.84	91.33 ± 32.67	5.06 ± 1.26
	G*	90 (20.4)				
rs2239704	CC	103 (46.6)	0.82 ± 0.21	8.19 ± 5.30	86.43 ± 36.67	5.48 ± 2.02
(LTA)	AC	100 (45.3)	0.85 ± 0.21	7.90 ± 4.64	78.49 ± 34.64	5.57 ± 3.54
	AA	18 (8.1)	0.87 ± 0.20	7.34 ± 1.95	84.67 ± 39.6	5.12 ± 1.49
	A*	136 (30.8)				
rs909253	AA	60 (27.3)	0.82 ± 0.22	8.27 ± 5.59	76.46 ± 33.33	5.98 ± 4.09
(LTA)	GA	110 (50.0)	0.85 ± 0.19	7.52 ± 2.88	86.99 ± 39.72	5.24 ± 2.11
	GG	50 (22.7)	0.81 ± 0.24	8.74 ± 6.79	81.00 ± 30.18	5.44 ± 1.84
	G*	210 (47.7)				
rs1041981	CC	59 (27.1)	0.82 ± 0.22	8.30 ± 5.63	76.72 ± 33.59	6.00 ± 4.12
(LTA)	AC	108 (49.5)	0.85 ± 0.19	7.53 ± 2.90	87.13 ± 40.06	5.23 ± 2.13
	AA	51 (23.4)	0.82 ± 0.24	8.67 ± 6.74	80.74 ± 29.91	5.40 ± 1.84
	A*	210 (48.2)				
rs1799964	AA	139 (62.9)	0.86 ± 0.21	7.79 ± 4.40	81.73 ± 33.76	5.18 ± 1.86
(TNF-α)	GA	73 (33.0)	0.79 ± 0.21	8.49 ± 5.72	84.88 ± 41.94	6.04 ± 4.00
	GG	9 (4.1)	0.87 ± 0.10	7.01 ± 0.85	79.92 ± 22.86	5.61 ± 1.68
	G*	91 (20.6)				
rs1800629	GG	167 (77.0)	0.84 ± 0.21	7.84 ± 4.11	84.42 ± 37.74	5.55 ± 3.01
(TNF-α)	AG	48 (22.1)	0.83 ± 0.23	8.66 ± 6.97	77.53 ± 29.86	5.25 ± 1.91
	AA	2 (0.9)	0.87 ± 0.11	6.96 ± 0.90	97.21 ± 36.52	5.67 ± 1.84
	A*	52 (12.0)				
rs3093662	AA	212 (95.9)	0.84 ± 0.21	7.91 ± 4.68	83.09 ± 36.35	5.45 ± 2.80
(TNF-α)	GA	9 (4.1)	0.77 ± 0.25	9.79 ± 7.01	71.93 ± 26.40	6.21 ± 2.26
	GG	0 (0.0)	—	—	—	—
	G*	9 (2.0)				
**Men**						
rs2794520	AA	78 (31.3)	0.86 ± 0.24	8.39 ± 6.42	101.30 ± 35.40	5.25 ± 2.41
(CRP)	GA	119 (47.8)	0.88 ± 0.24	7.76 ± 3.91	104.90 ± 39.04	5.37 ± 3.96
	GG	52 (20.9)	0.88 ± 0.18	7.50 ± 3.45	103.90 ± 37.00	4.95 ± 1.50
	G*	223 (44.8)				
rs1205	AA	78 (31.3)	0.86 ± 0.24	8.39 ± 6.42	101.30 ± 35.4	5.25 ± 2.41
(CRP)	GA	118 (47.4)	0.88 ± 0.23	7.67 ± 3.89	105.20 ± 38.9	5.35 ± 3.97
	GG	53 (21.3)	0.87 ± 0.18	7.59 ± 3.45	103.50 ± 36.58	4.96 ± 1.49
	G*	224 (45)				
rs1130864	GG	224 (91.1)	0.87 ± 0.22	7.87 ± 4.74	102.90 ± 37.3	5.27 ± 3.23
(CRP)	AG	21 (8.5)	0.9 ± 0.31	8.36 ± 5.54	111.70 ± 39.85	5.06 ± 1.71
	AA	1 (0.4)	1.11 ± 0.00	5.41 ± 0.00	104.40 ± 0.00	4.06 ± 0.00
	A*	23 (4.7)				
rs1800947	CC	212 (84.5)	0.88 ± 0.22	7.84 ± 4.84	103.50 ± 37.26	5.19 ± 3.20
(CRP)	GC	38 (15.1)	0.84 ± 0.24	8.25 ± 4.39	105.80 ± 37.35	5.60 ± 2.61
	GG	1 (0.4)	0.95 ± 0.00	6.31 ± 0.00	62.66 ± 0.00	4.31 ± 0.00
	G*	40 (8.0)				
rs3093059	AA	165 (65.7)	0.88 ± 0.23	7.86 ± 5.04	103.20 ± 35.91	5.35 ± 3.54
(CRP)	GA	78 (31.1)	0.85 ± 0.23	8.08 ± 4.38	103.30 ± 39.17	5.06 ± 2.11
	GG	8 (3.2)	0.89 ± 0.16	6.95 ± 1.21	116.60 ± 46.35	4.93 ± 1.3
	G*	94 (18.7)				
rs2239704	CC	111 (44.2)	0.87 ± 0.24	7.99 ± 4.77	101.30 ± 36.17	5.09 ± 2.01
(LTA)	AC	107 (42.6)	0.88 ± 0.22	7.96 ± 5.33	101.80 ± 36.68	5.53 ± 4.27
	AA	33 (13.2)	0.85 ± 0.18	7.35 ± 1.99	117.70 ± 40.56	4.88 ± 0.97
	A*	173 (34.5)				
rs909253	AA	76 (30.3)	0.87 ± 0.16	7.19 ± 1.72	109.10 ± 39.19	4.83 ± 1.13
(LTA)	GA	123 (49.0)	0.89 ± 0.25	8.22 ± 6.01	100.10 ± 34.79	5.60 ± 4.24
	GG	52 (20.7)	0.84 ± 0.24	8.17 ± 4.42	104.20 ± 39.71	5.02 ± 1.44
	G*	227 (45.2)				
rs1041981	CC	75 (30.2)	0.87 ± 0.17	7.20 ± 1.73	108.80 ± 39.41	4.83 ± 1.14
(LTA)	AC	122 (49.2)	0.89 ± 0.25	8.23 ± 6.03	100.00 ± 34.95	5.60 ± 4.25
	AA	51 (20.6)	0.85 ± 0.24	8.16 ± 4.46	104.20 ± 39.71	5.02 ± 1.46
	A*	224 (45.2)				
rs1799964	AA	155 (62)	0.87 ± 0.23	8.08 ± 5.12	105.50 ± 38.68	5.37 ± 3.63
(TNF-α)	GA	87 (34.8)	0.88 ± 0.22	7.65 ± 4.32	102.50 ± 34.68	5.10 ± 2.17
	GG	8 (3.2)	0.88 ± 0.16	7.00 ± 1.39	88.96 ± 34.94	4.65 ± 0.63
	G*	103 (20.6)				
rs1800629	GG	206 (83.7)	0.87 ± 0.23	7.97 ± 4.99	102.20 ± 37.38	5.26 ± 3.22
(TNF-α)	AG	40 (16.3)	0.90 ± 0.21	7.18 ± 2.53	113.70 ± 33.27	4.88 ± 1.69
	AA	0 (0.0)	—	—	—	—
	A*	40 (8.1)				
rs3093662	AA	241 (96.0)	0.87 ± 0.23	7.90 ± 4.83	104.10 ± 37.49	5.25 ± 3.16
(TNF-α)	GA	10 (4.0)	0.82 ± 0.19	7.79 ± 2.40	91.62 ± 29.01	5.25 ± 1.65
	GG	0 (0.0)	—	—	—	—
	G*	10 (2.0)				

Data are presented as n (%) for genotypes and alleles. TUG: Timed up-and-go; WaLP: weight-adjusted leg press.

*Minor allele.

a: All P > 0.05 from Hardy-Weinberg Equilibrium test.

Since TNF-α is one of the proinflammatory cytokines that initiates and regulates the cytokine cascade during an inflammation response, the bi-locus interactions of TNF-α SNPs with CRP and LTA SNPs were explored by considering this biological interactions. We didn’t identify any significant gene-gene interaction. On the contrary, we detected significant interactions between physical activity with CRP rs2794520, rs1205, and rs3093059; LTA rs909253 and rs1041981; and TNF-α rs1799964 for TCS in women after covariate adjustment (all P < 0.05) (Table 3). In men, significant interactions between physical activity with CRP rs2794520, rs1205, and rs3093059; and LTA rs909253 and rs1041981 for TUG; with CRP rs2794520, rs1205, rs1130864, and rs3093059; and LTA rs909253 and rs1041981 for walking speed; and with TNF-α rs3093662 for waLP after covariate adjustment (all P < 0.05).

**Table 3:** Significant gene–physical activity interactions on TUG, walking speed, waLP, and TCS.

Gene	SNP	P for interaction			
		TUG	Walking speed	waLP	TCS
**Women**					
CRP	rs2794520				0.017
CRP	rs1205				0.017
CRP	rs3093059				0.011
LTA	rs909253				0.017
LTA	rs1041981				0.017
TNF-α	rs1799964				0.017
**Men**					
CRP	rs2794520	0.006	0.042		
CRP	rs1205	0.006	0.042		
CRP	rs1130864		0.022		
CRP	rs3093059	0.013	0.022		
LTA	rs909253	0.008	0.030		
LTA	rs1041981	0.008	0.030		
TNF-α	rs3093662			0.033	

Additionally adjustment of age, BMI, smoking, drinking, and number of disease history.

We explored these significant interactions by estimating the adjusted means in lower extremity performance according to physical activity and CRP, LTA, and TNF-α genotype (minor–major/minor–minor and major–major genotypes) (Supplementary Figures 1–3). In women, we found that the adjusted means of TCS for GA/GG genotype of CRP rs2794520, rs1205, and rs3093059; LTA rs909253 and rs1041981; and TNF-α rs1799964 were significantly higher than AA genotype in elders without physical activity (adjusted means for minor–major/minor–minor vs. major–major genotypes: 6.81 vs. 9.08; 6.81 vs. 9.09; 5.80 vs. 8.70; 6.85 vs. 9.58; 6.86 vs. 9.57; and 9.53 vs. 6.73 [sec], respectively), but not in elders with physical activity. In men, the adjusted means of TUG for GA/GG genotype of CRP rs2794520, rs1205, and rs3093059; and LTA rs909253 and rs1041981 were significantly higher than AA genotype in elders without physical activity (9.66 vs. 14.19; 9.64 vs. 14.17; 9.05 vs. 12.47; 7.61 vs. 12.75; and 7.62 vs. 12.71 [sec], respectively), but not in elders with physical activity. For waLP, we observed a higher adjusted mean for TNF-α rs3093662 GA/GG genotype compared with that of AA genotype in elders without physical activity (157.55 vs. 83.10 [%]), but the opposite direction of effect in elders with physical activity (77.97 vs. 106.01 [%]). As for walking speed, GA/GG genotype of CRP rs2794520, rs1205, and rs3093059 were associated with a significantly higher means than AA genotype in elders without physical activity (0.78 vs. 0.65; 0.78 vs. 0.65; 0.85 vs. 0.68; and 0.69 vs. 0.85 [m/sec], respectively), but not in elders with physical activity except CRP rs3093059 with a significantly higher adjusted mean for AA genotype relative to GA/GG genotype (0.84 vs. 0.92 [m/sec]). In addition, the adjusted mean of AG/AA genotype for CRP rs11308649 was higher than that of GG genotype in elders with physical activity (1.02 vs. 0.89 [m/sec]). As for LTA gene on walking speed, we found that AA genotype of LTA rs909253 and CC genotype of LTA rs1041981 were associated with a high mean than their corresponding major-major genotype in elders without physical activity (both 0.69 vs. 0.85 [m/sec]), but not in elders with physical activity.

Multiple linear regression analyses were performed on haplotypes in the CRP gene and in the TNF-α/LTA genes according to gender. The haplotypes with a frequency of >5% were retained for analysis, and we identified five haplotypes of CRP genes and three of LTA genes in women for analysis (Table 4). Three haplotypes of CRP gene were prevalent, and the other two CRP haplotypes were less common with estimated frequencies of 9.73% and 5.94%. Elderly women who carried the G-G-A-C-A CRP haplotype displayed significantly slower walking speed and lower waLP after covariate adjustment (β = −0.08 m/s and −17.49%, respectively; both P < 0.05).

**Table 4:** The effect of CRP haplotypes on walking speed and waLP in women.

SNP	Haplotype	% of each haplotype	Walking speed (m/sec)				waLP (%)			
			Crude model		Adjusted model^a^		Crude model		Adjusted model^a^	
			β (SE)	p-value	β (SE)	p-value	β (SE)	p-value	β (SE)	p-value
CRP rs2794520-rs1205-rs1130864-rs1800947-rs3093059	A-A-G-C-A	47.10	reference	—	reference	—	reference	—	reference	—
	G-G-G-C-G	20.10	−0.01 (0.02)	0.643	−0.01 (0.02)	0.539	3.43 (4.83)	0.477	1.79 (4.54)	0.693
	A-A-G-G-A	9.73	0.01 (0.03)	0.737	−0.02 (0.03)	0.448	1.70 (6.17)	0.783	−3.15 (5.83)	0.590
	G-G-A-C-A	5.94	−0.08 (0.04)	0.048	−0.08 (0.04)	0.036	−15.57 (8.34)	0.062	−17.49 (7.85)	0.026
	G-G-G-C-A	16.90	−0.02 (0.03)	0.518	−0.03 (0.03)	0.247	−3.97 (5.39)	0.461	−8.31 (5.19)	0.110

β = estimated coefficient of regression model; SE = standard error.

a: Adjustment of age, BMI, physical activity, smoking, drinking, and number of disease history.

## Discussion

Inflammation may play an essential role in the physical performance of the elderly. In the present study, we attempted to examine the evidence for gene–gene and gene–physical activity interactions between the five SNPs in the *CRP* gene and six SNPs in the LTA–TNF-α gene related to lower extremity performance in Chinese elders in Taiwan. The results showed that physical activity was generally interactive with SNPs in the CRP, LTA, and TNF-α genes on TCS in the elderly women. Moreover, physical activity was interactive with SNPs in the CRP and LTA genes on TUG and with SNPs in CRP, LTA, and TNF-α genes on walking speed in elderly men. These findings could provide valuable information toward delineating the genetic mechanisms underlying inflammation for lower extremity performance in the elderly.

Prior epidemiologic studies that explored the relationship between inflammation and a decline in muscular mass and strength focused on inflammatory markers, instead of inflammatory genes. A previous study explored the relationship between plasma concentrations of TNF-α and muscular mass and strength, including appendicular muscular mass, thigh muscular area, grip strength, and knee extensor strength, in elderly men and women with good physical function. The results showed that an elevated level of TNF-α was associated with reduced muscular mass and strength^43^. This association, in general, was consistent across the gender and race groups, except for white men. Another study examined the association between physical performance and plasma levels of CRP, and TNF-α in the older participants of InCHIANTI study in Italy. The results showed that a higher level of CRP was associated with handgrip strength and composite physical performance score, including walking speed test, chair-stand test, and standing balance test^14^. Another study investigated the association between CRP concentration and the activities of daily living (ADL) among middle-aged and older persons in Indonesia^45^. This study identified a significant association between the high level of CRP and lower ADL among older persons. This study also provided additional findings on the significant association between CRP and ADL in middle-aged persons. A recent multi-nation study conducted in the five cities of Natal (Brazil), Manizales (Colombia), Rirana (Albania), Saint-Hyacinthe (Quebec, Canada), and Kingston (Ontario, Canada) reported significant graded associations between low CRP level and faster gait speed, as well as shorter time to rise from a chair^44^. In addition, significant associations between CRP and impaired balance and poor physical performance assessed by the Short Physical Performance Battery were observed^44^. Our study findings are consistent with those from the latter three studies, demonstrating that higher serum CRP level was associated with poorer physical function [17, 58, 59]. In our study, we did not detect significant association between TNF-α level and lower extremity performance, which were also consistent with the findings of the first two prior studies [57, 17].

Previous epidemiological genetic studies of CRP and TNF/LTA genes principally focused on muscular mass, muscular strength, or handgrip strength^48–51^. After extensive review of literature, no prior study that examined the effect of inflammatory polymorphisms on lower extremity performance was found. However, one previous study reported on the associations between TNF promoter polymorphisms and muscle phenotypes in 1050 participants aged 20 years and older under the Baltimore Longitudinal Study of Aging^47^. The authors detected five TNF promoter SNPs, namely, rs1799964, rs1799724, rs1800629, rs361525, and rs1800630. The results showed that rs1799964 and rs1800630, putative high-expression alleles, were associated with lower arm muscular mass. Moreover, rs1799964 was linked with appendicular skeletal muscular mass. In addition, carriers of the haplotype rs1799964 C- rs1800630 A- rs1799724 C- rs1800629 G- rs361525 G exhibited lower arm muscular mass and lower trunk muscular mass, compared with non-carriers. Although muscular mass and strength have been reported as predictors of physical performance^52^ or incident mobility limitation^53^, the percentage of the variance in performance explained by strength alone was always low (<20%). Thus, the relationship between inflammatory genetic variation and lower extremity performance is worthy of investigation. In our previous study, TNF-α rs1799964 interaction with LTA rs909253 or rs1041981 in women significantly affected serum hs-CRP level but not TNF-α level^46^. Our present study findings indicated that three variants involved in these two interactions also interacted with physical activity on TUG, walking speed, and waLP in men. Evidence from previous studies suggested that physical activity increases muscular strength and physical performance^54, 55^ by inducing an anti-inflammatory effect to protect against CRP and TNF-α induced aging^56^. The gene–physical activity interactions detected in our study suggest that physical activity intervention program may improve poor lower extremity performance.

Our study is limited by its relatively small sample size particularly for exploring gene–gene interactions. Therefore, we may not detect important polymorphisms associated with lower extremity performance because of the limited statistical power for stratified analysis. Future studies with larger number of elders are needed. Another limitation of this study is the few polymorphisms analyzed such that whether the effect observed indicated that these polymorphisms were true susceptibility allele or were due to LD to the “true” susceptibility allele remain unknown.

Our study had important strengths. This study was the first to assess the potential gene–gene interactions between the five polymorphisms in the CRP gene and six in the TNF-LTA genes and lower extremity performance, as well as interactions between these 11 variants, with the physical activity of Chinese elders living in community. Second, our study subjects were from a representative sample of Chinese elderly population. Therefore, our generalized results can be applied to other Chinese elderly populations. Third, the measurement of genotypes had stringent quality controls.

In conclusion, the current study showed that one variant (rs1799964) in the TNF-α gene, three variants (rs2794520, rs1205, and rs3093059) in the CRP gene and three variants (rs909253, rs1041981, and rs2239704) in the LTA gene interacted with physical activity in both elderly men and women, suggesting that environmental factors may modify these genotypic effects. These findings will facilitate the delineation of genomic mechanism by which physical activity modifies the effect of these polymorphisms. Our study provides new insights for genetic screening test and physical activity intervention programs in elders.

## Methods

### Study participants

This population-based cross-sectional study included 472 unrelated elderly participants (221 women and 251 men aged 65 years and over) of the Taichung Community Health Study for Elders (TCHS-E). Study participants were recruited from Taichung city in Taiwan, and the recruitment process has been described in detail in our previous publication^42, 57^. All elders aged 65 years and older who resided in eight administrative neighborhoods of Taichung city were specified as our study subjects and invited to participate in the TCHS-E. We selected elders residing in these eight administrative neighborhoods as our study subjects to facilitate future follow-up activities. In addition, the age and gender distributions of elders in these neighborhoods are similar to those of the Taichung population and Taiwan populations. A total of 2,750 eligible elderly resided in these neighborhoods during the time of the study, but only 1,347 agreed to participate for an overall response rate of 49.0%. Among these subjects, the genetic specimens of 480 subjects were sent for genotyping. Eight subjects with incomplete data were eliminated from data analysis. All subjects provided their informed consent to participate in the study. This study was approved by the Human Research Committee of China Medical University Hospital and all methods were performed in accordance with the relevant guidelines and regulations.

### Measurements of performance tests

The tests included measures of walking speed, timed up-and-go (TUG) test, timed chair stands (TCS), and weight-adjusted leg press (waLP). The participants underwent all physical performance tests under the instructions of physical therapists. For the walking test, the participants were asked to walk 5 m at their usual gait speed. The walking speed was calculated as 5 m divided by the recorded time. Three trials were performed, and the average speed was used for data analysis. The TUG test required the participant to stand up from a sitting position, walk 3 m from that position, walk back to the chair, and sit down as fast as possible^58^. The test was repeated thrice, and the shortest time elapsed was recorded for data analysis. The participants in the TCS test were asked to fold their arms across their chest and to sit firmly in a chair, as well as to stand up and sit down thrice, and time elapsed was recorded^59^. The participants were not allowed to use their hands for support during the test, and the test was repeated thrice. The shortest time elapsed was used for analysis. For waLP, submaximal leg press strength (a maximum of 10–15 repetitions) was measured by a leg press machine (AURA G3-S70, Matrix Fitness System, USA). Then, one-repetition maximum leg press strength was then estimated by the Brzycki formula^60^. WaLP was calculated by dividing the corresponding results by the weight of the participant.

### Other measurements

Blood was drawn with minimal trauma from an antecubital vein in the morning after a 12-h overnight fasting and was sent for analysis within 4 h of blood collection. Sociodemographic and lifestyle characteristics of each participant were collected using a self-administered questionnaire. A participant’s physical activity for leisure time was measured by the different kinds of physical activities each person participated in through a checklist of 34 items. Each item contains questions on the average time spent on each activity per occasion and the average number of the activity performed per week during the past six months prior to participation. Participants with regular physical activity were defined as those who participated in regular leisure-time activities for at least 30 min once per week during the preceding six months. An autoanthropometer (super-view, HW-666) was used to measure the weight and height of a shoeless subject wearing light clothing. BMI (*kg/m* ^*2*^) was calculated by dividing the weight of a subject in kilogram by the square of the height in meter.

### SNP selection and genotyping

We selected 11 tag-SNPs, consisting of five CRP tag-SNPs (rs1205, rs1130864, rs1800947, rs2794520, and rs3093059), three LTA tag-SNPs (rs909253, rs1041981, and rs2239704), and three TNF-α tag-SNPs (rs3093662, rs1800629, and rs1799964), for genotyping based on prior studies for the Han population from Beijing, China in the International HapMap Project. A commercial kit (QIAamp DNA Blood Kit; Qiagen, Chatsworth, CA, USA) was used to extract genomic DNA. The GoldenGate assay from Illumina Inc. (San Diego, CA, USA) was used to genotype all tag-SNPs. We evaluated in advance the quality of genomic DNA specimen to improve the success rate of genotyping. An exact χ^2^ goodness of fit test was adopted to evaluate the HWE in the men and women for each tag-SNP using PLINK software (v1.07, http://pngu.mgh.harvard.edu/purcell/plink). A threshold of two-tailed P < 0.05 was applied to indicate departures from HWE.

### Statistical analysis

Simple descriptive analyses, such as mean, standard deviation, proportion, χ^2^ test, and t-test were employed to compare differences in demographic, lifestyle characteristics, and physical performance, such as age, BMI, walking speed, TUG, waLP, and TCS, between men and women when appropriate. At first, the main effects of genotype for eleven SNPs were examined by analysis of variance (ANOVA). Then gene–gene interactions on the physical performance measures were assessed by multiple linear regression analyses adjusted for age, physical activity, BMI, drinking habits, smoking habits, and the number of disease history. We derived an indicator with major-major genotype as the reference and minor-major/minor-minor as indicator for each SNP. The interactions between two SNPs were evaluated with the indicators measuring the main effects of these two SNPs and the product terms of indicators of these two SNPs being entered into the model. Using the same approach, the gene-physical activity interactions had been evaluated. Finally, interaction plots were presented for those analysis with significant interactions. To account for multiple testing, we reported p-values using false discovery rate (FDR) method, a linear step up adjustment, to control for false discovery rate. For each outcomes including walking speed, TUG, waLP, and TCS, a total of eleven tests were accounted to obtain the reported FDR p-values. Furthermore, the haplotypes in this sample were analyzed separately for each gene at a frequency >5%. The significance level was set to a two-sided P < 0.05. All statistical analyses, including univariate analysis of simple descriptive statistics, bivariate analysis of t-test, χ^2^ test, and ANOVA, multiple linear regression, and FDR method, were stratified by gender using Statistical Analysis System software (v9.4, SAS Institute Inc., Cary, NC, USA).

## Electronic supplementary material

Supplementary Figure 1-3
