# Supplementary material for: Gene–physical activity interactions in lower extremity performance: inflammatory genes CRP, TNF-α, and LTA in community-dwelling elders
Source: Sci Rep. 2017 Jun 15;7:3585. doi: 10.1038/s41598-017-03077-1 (PMC5472589; doi:10.1038/s41598-017-03077-1)
Supplement: Supplementary file 1 — Supplementary Figure 1-3 [file 41598_2017_3077_MOESM1_ESM.pdf]

**Supplementary information**

Gene–physical activity interactions in lower extremity performance: inflammatory genes CRP, TNF-α, and LTA in community-dwelling elders

Chiu-Shong Liu[1,2#], Tsai-Chung Li[3,4, #], Chia-Ing Li[1,5,], Li-Na Liao[1,] , Chuan-Wei Yang[5,6], Chih-Hsueh Lin[1,2], Nai-Hsin Meng[1,7], Wen-Yuan Lin[1,2], Sung-Lin Hu[2], Jen-Hao Hsiao[8], Fang-Yang Wu[3], and Cheng-Chieh Lin[1,2*]

1. School of Medicine, College of Medicine, China Medical University, Taichung, Taiwan
2. Department of Family Medicine, China Medical University Hospital, Taichung, Taiwan
3. Department of Public Health, College of Public Health, China Medical University, Taichung, Taiwan
4. Department of Healthcare Administration, College of Medical and Health Sciences, Asia University, Taichung, Taiwan
5. Department of Medical Research, China Medical University Hospital, Taichung, Taiwan
6. Ph.D. Program for Aging, College of Medicine, China Medical University, Taichung, Taiwan
7. Department of Physical Medicine and Rehabilitation, China Medical University Hospital, Taichung, Taiwan
8. Bioinformatics and Biostatistics Core, Center of Genomic Medicine, National Taiwan University, Taipei, Taiwan

[#]Equal contribution as the first author

***Corresponding authors:** Cheng-Chieh Lin (cclin@mail.cmuh.org.tw)

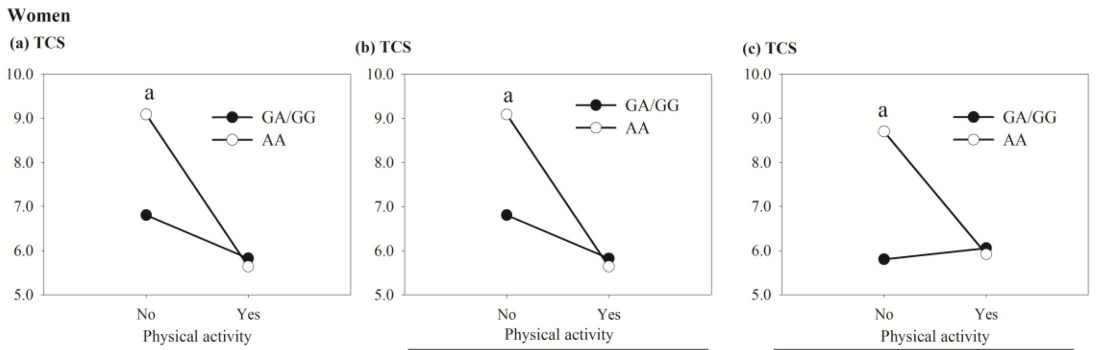


| Physical activity | CRP rs2794520 | |
|---|---|---|
| | GA/GG | AA |
| No | 29 | 15 |
| Yes | 115 | 52 |

| Physical activity | CRP rs1205 | |
|---|---|---|
| | GA/GG | AA |
| No | 29 | 15 |
| Yes | 114 | 52 |

| Physical activity | CRP rs3093059 | |
|---|---|---|
| | GA/GG | AA |
| No | 14 | 30 |
| Yes | 60 | 107 |

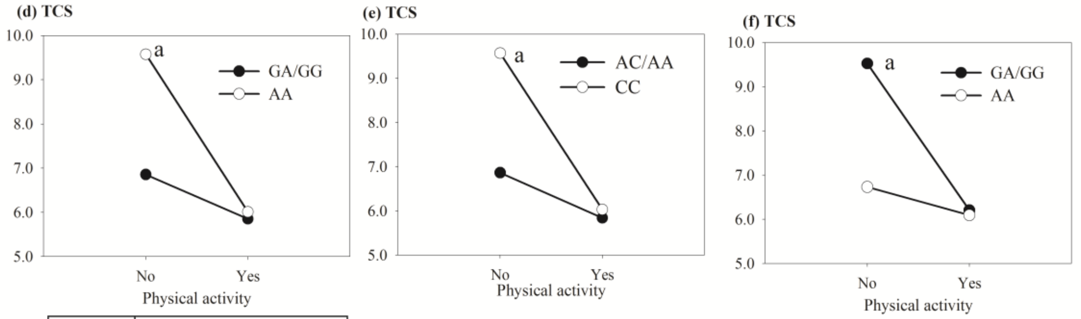


| Physical activity | LTA rs909253 | |
|---|---|---|
| | GA/GG | AA |
| No | 30 | 14 |
| Yes | 120 | 46 |

| Physical activity | LTA rs1041981 | |
|---|---|---|
| | AC/AA | CC |
| No | 28 | 14 |
| Yes | 121 | 45 |

| Physical activity | TNF-α rs1799964 | |
|---|---|---|
| | GA/GG | AA |
| No | 19 | 25 |
| Yes | 61 | 106 |

**Supplementary Figure 1.** Adjusted means of TCS (sec) in SNP according to status of physical activity and SNP for significant gene–physical activity interaction after adjustment for age, BMI, smoking, drinking, and number of disease history in women.

a: Significant comparisons of adjusted means between minor–major/minor–minor genotypes and major–major genotype in elders without physical activity ($P<0.05$).

b: Significant comparisons of adjusted means between minor–major/minor–minor genotypes and major–major genotype in elders with physical activity ($P<0.05$).

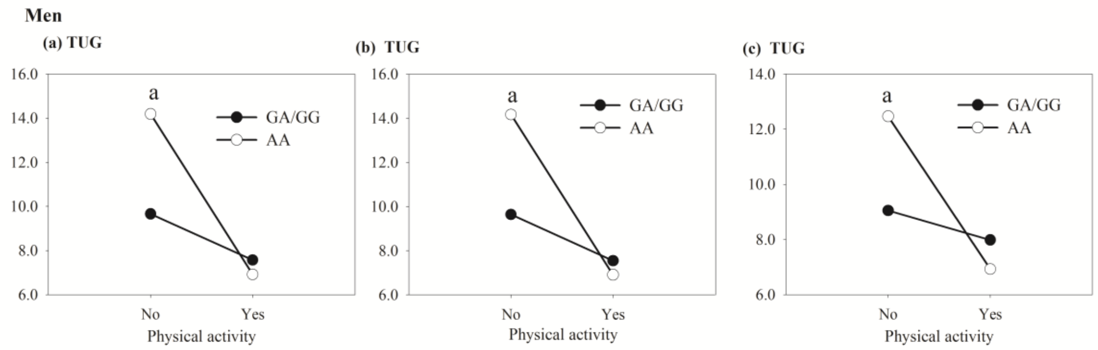


| Physical activity | CRP rs2794520 | |
|---|---|---|
| | GA/GG | AA |
| No | 21 | 17 |
| Yes | 146 | 61 |

| Physical activity | CRP rs1205 | |
|---|---|---|
| | GA/GG | AA |
| No | 21 | 17 |
| Yes | 146 | 61 |

| Physical activity | CRP rs3093059 | |
|---|---|---|
| | GA/GG | AA |
| No | 10 | 28 |
| Yes | 74 | 135 |

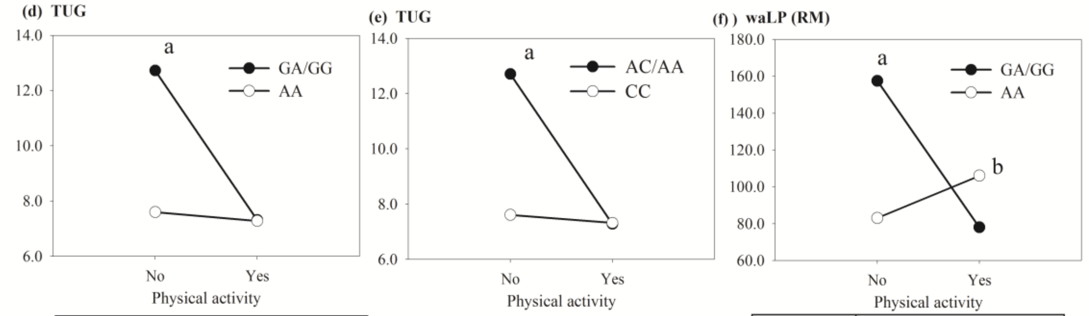


| Physical activity | LTA rs909253 | |
|---|---|---|
| | GA/GG | AA |
| No | 30 | 8 |
| Yes | 142 | 67 |

| Physical activity | LTA rs1041981 | |
|---|---|---|
| | AC/AA | CC |
| No | 30 | 8 |
| Yes | 140 | 66 |

| Physical activity | TNF-α rs3093662 | |
|---|---|---|
| | GA/GG | AA |
| No | 1 | 30 |
| Yes | 8 | 191 |

**Supplementary Figure 2.** Adjusted means of TUG (sec) and waLP (%) in SNP according to status of physical activity and SNP for significant gene–physical activity interaction after adjustment for age, BMI, smoking, drinking, and number of disease history in men.

a: Significant comparisons of adjusted means between minor–major/minor–minor genotypes and major–major genotype in elders without physical activity ($P<0.05$).

b: Significant comparisons of adjusted means between minor–major/minor–minor genotypes and major–major genotype in elders with physical activity ($P<0.05$).

**Men**

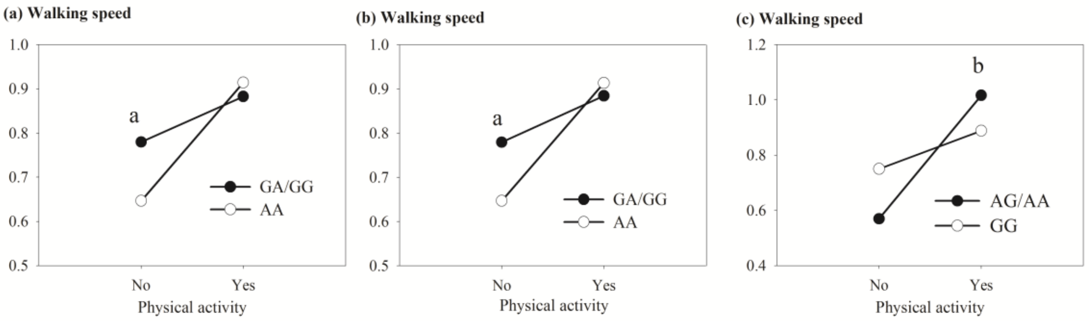


| Physical activity | CRP rs2794520 | |
|---|---|---|
| | GA/GG | AA |
| No | 21 | 17 |
| Yes | 145 | 61 |

| Physical activity | CRP rs1205 | |
|---|---|---|
| | GA/GG | AA |
| No | 21 | 17 |
| Yes | 145 | 61 |

| Physical activity | CRP rs11308649 | |
|---|---|---|
| | AG/AA | GG |
| No | 5 | 33 |
| Yes | 15 | 188 |

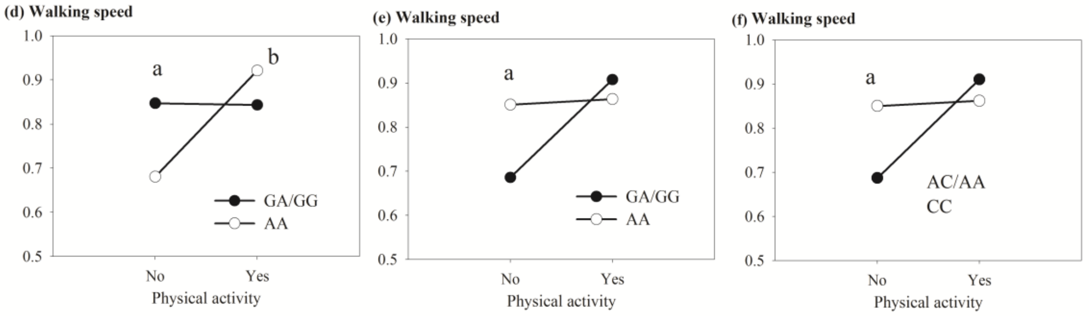


| Physical activity | CRP rs3093059 | |
|---|---|---|
| | GA/GG | AA |
| No | 10 | 28 |
| Yes | 73 | 135 |

| Physical activity | LTA rs909253 | |
|---|---|---|
| | GA/GG | AA |
| No | 30 | 8 |
| Yes | 141 | 67 |

| Physical activity | LTA rs1041981 | |
|---|---|---|
| | AC/AA | CC |
| No | 30 | 8 |
| Yes | 139 | 66 |

**Supplementary Figure 3**. Adjusted means of walking speed (m/sec) in SNP according to status of physical activity and SNP for significant gene–physical activity interaction after adjustment for age, BMI, smoking, drinking, and number of disease history in men.

a: Significant comparisons of adjusted means between minor–major/minor–minor genotypes and major–major genotype in elders without physical activity ($P<0.05$).

b: Significant comparisons of adjusted means between minor–major/minor–minor genotypes and major–major genotype in elders with physical activity ($P<0.05$).
